# Novel deletion of exon 3 in *TYR *gene causing Oculocutaneous albinism 1B in an Indian family along with intellectual disability associated with chromosomal copy number variations

**DOI:** 10.1186/s12920-021-01152-1

**Published:** 2022-01-03

**Authors:** Somprakash Dhangar, Purvi Panchal, Jagdeeshwar Ghatanatti, Jitendra Suralkar, Anjali Shah, Babu Rao Vundinti

**Affiliations:** Department of Cytogenetics, National Institute of Immunohaematology (ICMR), 13th floor, new multistoried building, K.E.M Hospital campus, Parel, Mumbai, 400012 India

**Keywords:** Oculocutaneous albinism (OCA), Intellectual disability (ID), *TYR *gene, Chromosomal copy number variations, Complex molecular diagnosis, Next generation sequencing (NGS), Multiplex ligation-dependent probe amplification (MLPA)

## Abstract

**Background:**

Oculocutaneous albinism (OCA) is an autosomal recessive disorder characterized by hypo-pigmentation of skin, hair, and eyes. The OCA clinical presentation is due to a deficiency of melanin biosynthesis. Intellectual disability (ID) in OCA cases is a rare clinical presentation and appropriate diagnosis of ID is challenging through clinical examination. We report an Indian family with a rare co-inheritance of OCA1B and ID due to a novel *TYR* gene variant and chromosomal copy number variations.

**Methods:**

We have done a study on three siblings (2 males and 1 female) of a family where all of them presented with hypopigmented skin, hair and eyes. The male children and their father was affected with ID. Targeted exome sequencing and multiplex ligation-dependent probe amplification analysis were carried out to identify the OCA1B and ID associated genomic changes. Further Array-CGH was performed using SurePrint G3 Human CGH + SNP, 8*60 K array.

**Results:**

A rare homozygous deletion of exon 3 in *TYR *gene causing OCA1B was identified in all three children. The parents were found to be heterozygous carriers. The Array-CGH analysis revealed paternally inherited heterozygous deletion (1.9 MB) of 15q11.1-> 15q11.2 region in all three children. Additionally, paternally inherited heterozygous deletion (2.6 MB) of 10q23.2-> 10q23.31 region was identified in the first male child; this may be associated with ID as the father and the child both presented with ID. While the 2nd male child had a denovo duplication of 13q31.1-> 13q31.3 chromosomal region.

**Conclusion:**

A rare homozygous *TYR *gene exon 3 deletion in the present study is the cause of OCA1B in all three children, and the additional copy number variations are associated with the ID. The study highlights the importance of combinational genetic approaches for diagnosing two different co-inherited disorders (OCA and ID). Hence, OCA cases with additional clinical presentation need to be studied in-depth for the appropriate management of the disease.

**Supplementary Information:**

The online version contains supplementary material available at 10.1186/s12920-021-01152-1.

## Background

Oculocutaneous albinism (OCA) is an autosomal recessive disorder that occurs due to deficiency of melanin biosynthesis resulting in generalized hypo-pigmentation of skin, eyes, and hair [1]. The incidence of OCA is not known; however, it varies in different regions of the world. Montoliu et al. reported the incidence of albinism is 1:17,000 in the Western population (North America & Europe). Federico et al. estimated the overall incidence of OCA was 1:17,000 to 1:20,000 [2, 3]. Based on the genetic etiology OCA (nonsyndromic) is classified into eight different subtypes (OCA1-7 and OA) [3–5]. The overlapping syndromic albinism such as Hermansky–Pudlak syndrome (HPS), Griscelli syndrome (GS), and Chediak–Higashi syndrome (CHS) have also been reported. Among all, OCA1 is the second most common sub-group of OCA categorized into two types; OCA1A (complete lack of Tyrosinase activity results in the absence of pigmentation in hair, skin, and eyes) and OCA1B (with residual Tyrosinase activity) [6]. OCA2 is commonly seen in African Americans, some Native American groups, and sub-Saharan Africans. The OCA type 3, specifically rufous Oculocutaneous albinism, is reported in Southern Africa [3]. The OCA type 4 is commonly reported in Japanese and Korean populations compared to other parts of the world [7].

The *TYR *gene is located on the 11q14 chromosome region, consisting of 5 exons encoding 529 amino acids of tyrosinase protein. This protein has dual enzymatic activity as it catalyzes the hydroxylation of tyrosine to dopaquinone in the melanin biosynthesis pathway and is also involved in the oxidation of L-DOPA [8]. The homozygous point mutation or large multiple exonic deletions in the *TYR *gene are responsible for OCA1 in humans and other mammals [9, 10].

Intellectual disability (ID) and psychotic illness in OCA1 is a rare occurrence; however, it is reported in a few cases of Cross-McKusick-Breen Syndrome (CMBS) [11]. Though the copy number changes are associated with developmental delay, the co-inheritance of ID and albinism has not been reported in the literature. Though the deletions of chromosome number 10 and 15 are reported in a few studies, none of these reports showed the involvement of the *TYR *gene [12–16]. This study describes a rare co-inheritance of OCA1B and ID in an Indian family, with a rare *TYR* gene variant and chromosomal copy number variations.

## Methods

### Study design

The study was designed to understand OCA1B inheritance in three siblings and parents of the family. The NGS study was done to identify variants associated with the disease, and copy number variations were identified using array- comparative genomic hybridization (array-CGH).

### Subjects and clinical description

The study was carried out in an Indian family with three children (2 males and 1 female) affected with albinism. The affected children were born of a consanguineous marriage. All the three siblings were presented with hypo-pigmentation of skin (few pigmented spots on hand and face), slight pigmentation on knee region, pigmented iris, brownish-red hair, and photophobia. The 1^st^ child was an 11-year old male, and his clinical examination revealed developmental delay, facial dysmorphism (bulbous nose and wide ears), and ID. The 2^nd^ child was an 8-year old female, had only albinism. She was intellectually normal. The 3^rd^ child (5-year old, male) had ID along with Attention Deficit-Hyperactivity Disorder (ADHD) (hyperactivity, unable to sit still, aggressive) and showed mild facial dysmorphism with low set ears and mild hypertelorism. Both male children had severe speech retardation, and all the siblings had skin burns due to hypo-pigmentation of the skin. The complete blood count, liver and kidney function tests were found to be normal. Brainstem evoked response audiometry (BERA) examination revealed a normal hearing pattern. The father was 21-years old, while the mother was 18-years at the time of first pregnancy. The clinical evaluation of the parents revealed no bad obstetric history. The father had a history of poor social behavior with delayed development at childhood and is currently unable to perform complex routine activities, and the mother is intellectually normal.

### Targeted exome sequencing (TES)

Peripheral blood samples were collected in EDTA vacutainers from all affected and non-affected family members. Genomic DNA was isolated from the peripheral blood leukocytes using a Qiagen Mini DNA isolation kit. The concentration was estimated using Qubit™. TES was performed with selective capture of the protein-coding regions of the genes responsible for OCA and other similar phenotype using Next Generation Sequencing (NGS). All the genes covered in the clinical exome assay have been screened for the given clinical indications. The libraries were sequenced to mean > 80-100X coverage on the Illumina sequencing platform.

### Sanger sequencing and in-silico validation

The Sanger Sequencing was performed to confirm the variations identified through NGS. The analysis of *TYR *and *RP1* genes variants were carried out by conventional PCR and Sanger Sequencing. The PCR reaction was carried out under the following conditions: Dream Taq Master Mix, 10 µM of primers, 100 ng of genomic DNA. Amplification was carried out in 25-μL volumes with initial denaturation of 95 °C for 2 min, 35 cycles: 95 °C for 30 s, annealing temperature for 30 s, and 72 °C for 45 s and final extension of 72 °C for 5 min (Table 1).

**Table 1:** Primer sequences and their annealing temperature used for PCR and Sanger sequencing

Primer name	Sequence 5′–3′	Base pair	Annealing temperature
*RP1* (Forward)	TCCAAGAAGAGGTAGAGGCT	20	55
*RP1* (Reverse)	ACTGTCAGGCCGATAGTCT	19	
*TYR* (Forward)	AGGGAACACAAATTGGCTCA	20	62
*TYR* (Reverse)	TCCTGCCTAATCCACCTTCTT	21	

Direct sequencing was performed with the BigDye® terminator cycle sequencing ready reaction kit on an ABI prism 3730xl automated genetic analyzer. *RP1* gene variant identified through NGS was confirmed with Sanger Sequencing. The PCR conditions were common for both the genes except annealing temperature (Table 1). The results obtained from Sanger Sequencing of genes were analyzed using BLASTN tools.

### Multiplex ligation-dependent probe amplification (MLPA) assay

MLPA was performed using a commercially available kit (Cat. No. P325-A3 OCA2; MRC Holland) to identify the deletions in the *TYR *gene. A total of 5 µl DNA sample was heat-denatured for 5 min at 98 °C. Samples were cooled down to room temperature. The hybridization master mix (3 µl) was added to DNA and incubated for 1 min at 95 °C, and then the probes were hybridized for 16 h at 60 °C. Thermo cycler temperature was lowered to 54 °C, and a 32 µl ligase-65 master mix was added and incubated for 15 min at 54 °C for ligation of hybridized probes. The ligase enzyme was heat-inactivated at 98 °C for 5 min and cooled down to room temperature; a 10 µl polymerase master mix was added at room temperature to amplify ligated probes. PCR condition was for 35 cycles (95°c 30 s, 60°c 30 s, 72°c 60 s, 72°c 20 min, 15°c pause). Fragment separation by capillary electrophoresis was performed using an automated sequencer (ABI 3730xl). Results were analyzed using Coffalyser software. Each MLPA probe consisted of two hemi-probes that bind to adjacent sites on the target sequence. Upon ligation and subsequent PCR amplification, each distinct MLPA probe (specific to distinct target regions) generated an amplicon with a unique length, were separated and quantified by capillary electrophoresis. Heterozygous deletions within target sequences would prevent efficient probe binding and give a 35–50% reduced relative peak area of the amplification product specific to that probe set. The copy number differences of various exons between test and control DNA samples were detected by analyzing the MLPA peak patterns.

### Array-comparative genomic hybridization (array-CGH)

Array-CGH was performed on DNA samples using SurePrint G3 Human CGH + SNP, 8*60 K array. The data was analyzed using CytoGenomics 5.0.0.38 (Agilent, Santa Clara, California, USA), and nomenclature of Copy Number Variations (CNVs) was done using ACMG guidelines [17]. All genomic positions were reported according to the human genome reference assembly (GRCh37).

### Bioinformatics analysis

The sequences obtained from TES were aligned to the human reference genome (GRCh37/hg19) using the BWA program and analyzed using Picard and GATK version 3.6 to identify the variants relevant to the clinical indication. The GATK practices framework was followed for the identification of variants. The VEP program against Ensembl release 87 human gene model was used to perform Gene annotation of variants. Human Genome databases (ClinVar, OMIM, and HGMD) were used to annotate identified variants. The effects of these variants were calculated using Mutation Taster2.

In silico amino acid conservation site was analyzed using Multiple Amino Acid Sequence Alignment software (https://www.ebi.ac.uk/Tools/msa/clustalo). The protein sequences *RP1 *derived from humans, chimpanzees, rhesus monkeys, dogs, horses, murine, and cattle were compared. The *RP1* protein functional analysis was done using online ScanPrositeExpasy software (https://prosite.expasy.org/scanprosite).

## Results

**Fig. 1:**
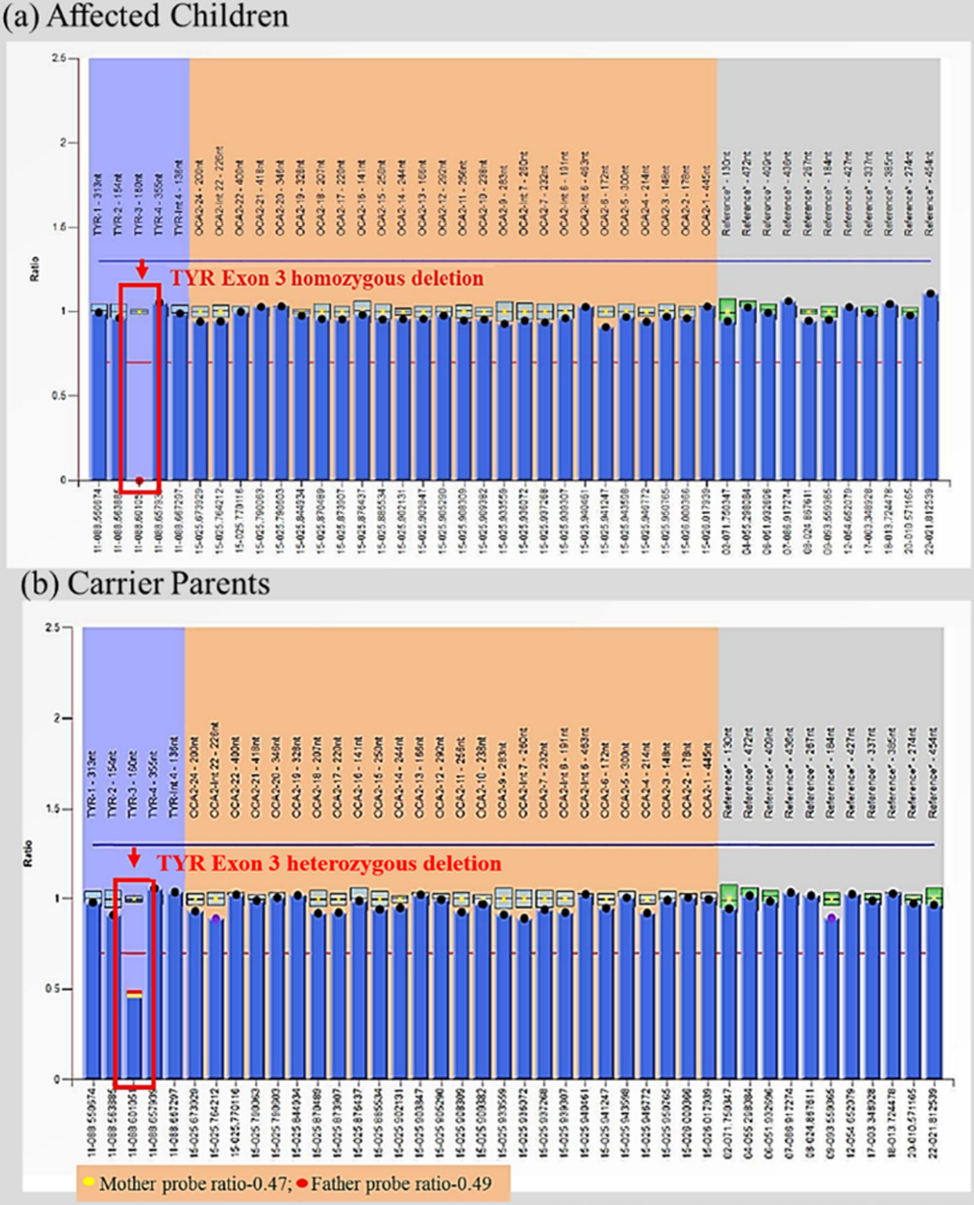
MLPA results showing *TYR* exon 3 deletion: (**a**) All 3 children of fourth-generation with probe ratio 0.00 (**b**) Carrier parents (Mother with probe ratio 0.47& father with probe ratio 0.49)

We have studied the family having three children affected with albinism & subjected to NGS (Additional file 1: Table S1). The NGS analysis failed to cover the *TYR* gene exon 3 region of affected subjects, otherwise usually well covered in healthy individuals. After multiple attempts, amplification of the *TYR *gene exon 3 in all three children using conventional PCR was failed. Multiplex Ligation-dependent Probe Amplification (MLPA) analysis revealed a novel large homozygous deletion (Probe ratio 0.00) of exon 3 of *TYR* gene in all the three children (Fig. 1a). A heterozygous deletion of exon 3 of the *TYR *gene was identified in the mother (Probe ratio 0.47) and father (Probe ratio 0.49) (Fig. 1b). The novel deletion detected in the present study was submitted in the ClinVar database (Accession no: SCV001448215).

**Fig. 2:**
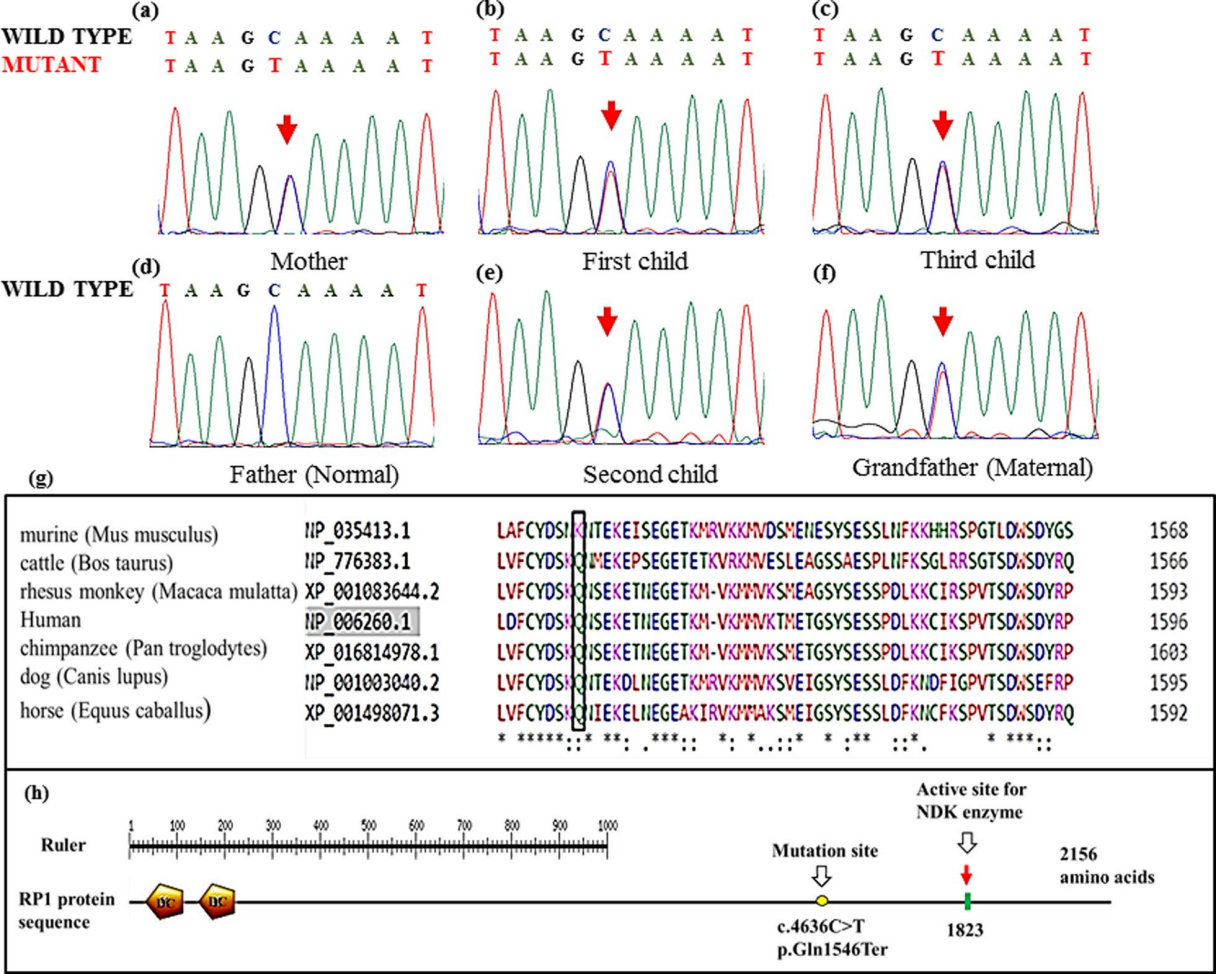
Chromatogram showing heterozygous *RP1* variation: (**a**) Mother (**b**) First child (**c**) Third child (**d**) Father (**e**) Second child (**f**) Maternal grandfather (**g**) Multiple amino acid sequence alignment analysis shows that the Amino acid p.Q1546 is moderately conserved among the primates (**h**) In silico *RP1* protein functional analysis illustrating the functional domain, enzyme active site and the variation site

The further analysis through NGS revealed a novel heterozygous nonsense variation in exon 4 of the *RP1* gene (chr8:55541078C > T; Depth: 55x) in the first child. Sanger Sequencing analysis confirmed the presence of *RP1* gene variation (p.Gln1546Ter) in all the three children, mother, and maternal grandfather (Fig. 2a–c, e, f). The *RP1* gene variant was absent in the father (Fig. 2d). The p.Gln1546Ter variation resulted in a stop codon and premature truncation of the protein at codon 1546 (p.Gln1546Ter; ENST00000220676.1). In silico prediction of the *RP1* gene variant (p.Gln1546Ter) was disease-causing (probably deleterious) by MutationTaster2. The evolutionary conservation of *RP1* variant p.Q1546Ter was determined by Multiple Amino Acid Sequence Alignment software. The *RP1* protein sequences derived from human, chimpanzee, rhesus monkey, dog, horse, murine, and cattle showed that the residue-1546 was moderately conserved among all the primates (Fig. 2g, h).

**Fig. 3:**
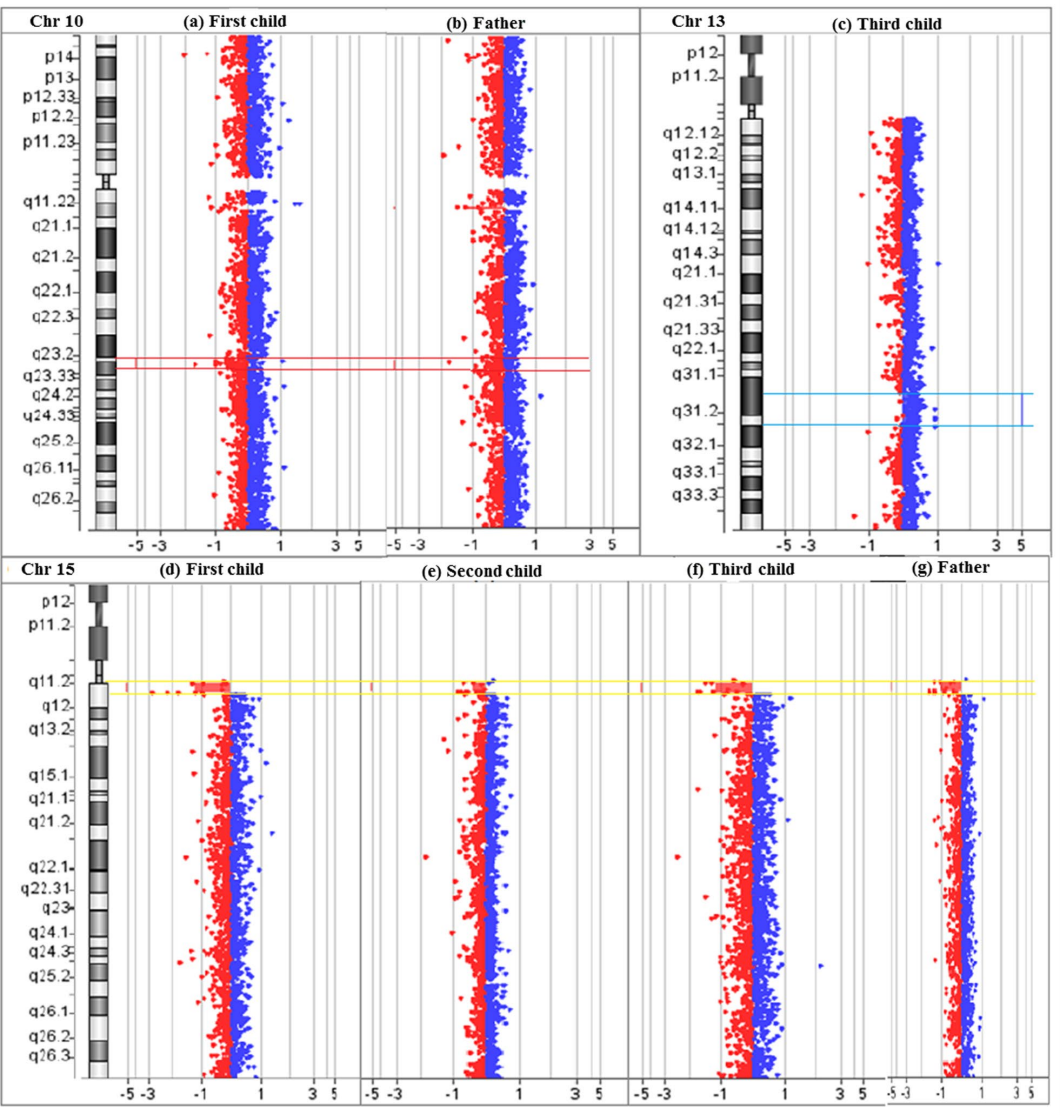
Array- CGH karyogram showing: **a**, **b** Deletion of chromosomal region 10q23.2q23.31 in first child and father (Left to right) **c** Denovo duplication of chromosomal region 13q31.1q31.3 in the third child (male) **d**–**g** Deletion of chromosomal region 15q11.1q11.2 in first, the second and third child and father (Left to right)

The array-CGH analysis revealed that the 1^st^ child had paternally inherited 2.6 MB deletion in chromosome 10q23 region {arr[GRCh37] 10q23.2q23.31 (88816618_91483662) × 1} and 1.9 MB deletion in chromosome 15q11 region {arr[GRCh37] 15q11.1q11.2 (20575646_22509254) × 1} (Fig. 3a, b, d, g). However, the second child (female) was identified with only one paternally inherited CNVs (15q11.1-> 15q11.2) {arr[GRCh37] 15q11.1q11.2(20575646_22509254) × 1} (Fig. 3e, g).The 3^rd^ child (male) was detected with paternally inherited CNVs (15q11.1-> 15q11.2) along with a denovo duplication (7.6 MB) of chromosome region 13q31.1-> 13q31.3 {arr[GRCh37] 13q31.1q31.3 (83199241_90822978) × 3} (Fig. 3c, f, g). Further, array CGH analysis in mother revealed no indication of CNVs.

**Fig. 4:**
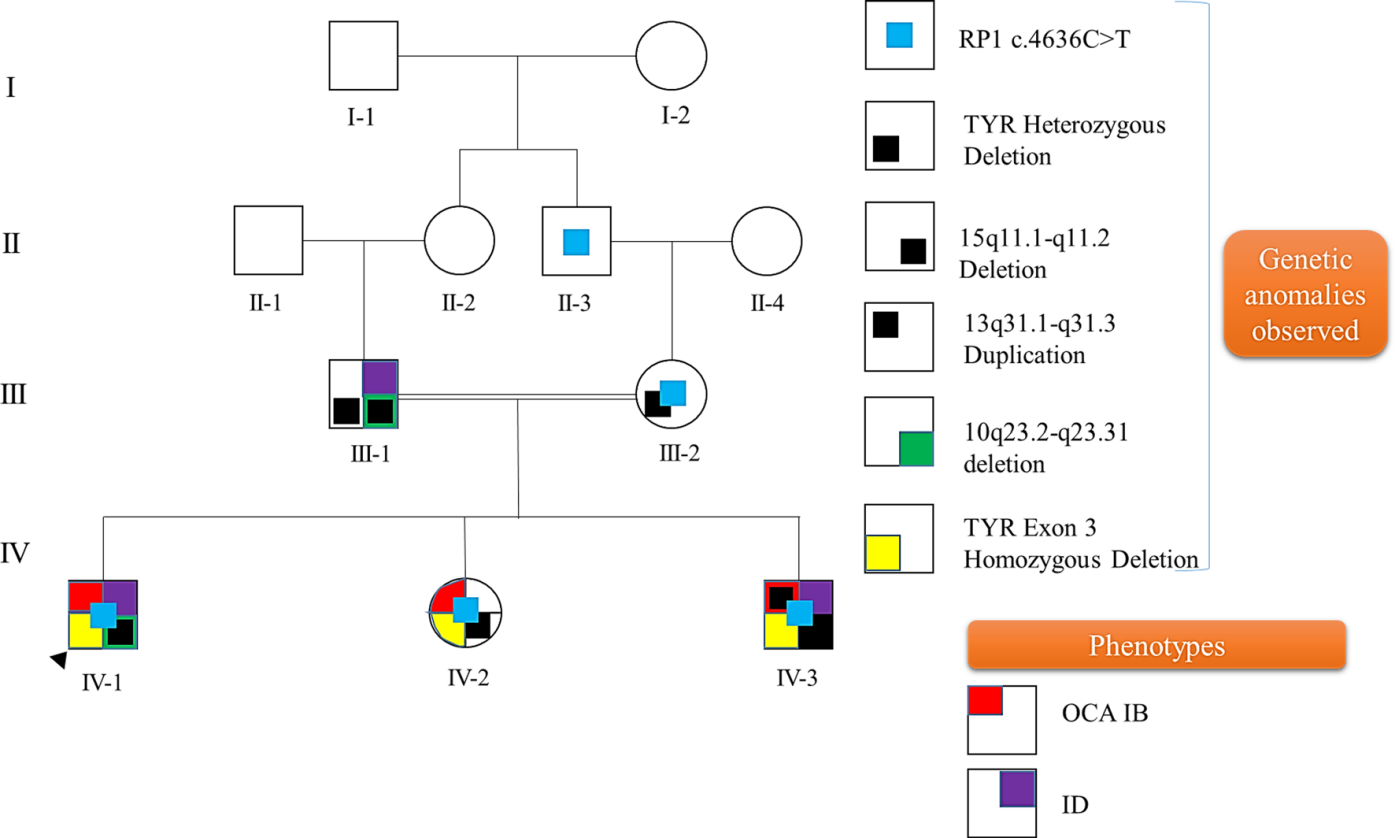
Family pedigree showing inheritance pattern of genetic anomalies and their phenotype

The pedigree analysis revealed autosomal recessive inheritance of OCA1B from the parents to all three children (Fig. 4). The ID in the first-born male child was found to be associated with 10q23.2-> 23.31 deletion inherited from father, while in the 3^rd^ child (male), ID was found to be associated with a denovo 13q31.1-> 13q31.3 duplication.

## Discussion

OCA is a genetically heterogeneous congenital disorder characterized by low or absence of pigmentation in the hair, skin, and eyes. The OCA1B (OMIM# 606952) occurs due to autosomal recessive inheritance of a pathogenic mutation in the *TYR* gene [3]. So far, more than 320 *TYR* gene mutations and polymorphisms, including large multiple exon deletions have been reported in the Human Gene Mutation Database (https://www.hgmd.cf.ac.uk/ac/index.php/) provided in the public domain by the Institute of Medical Genetics, Cardiff, Wales, UK) and the Albinism Database (http://albinismdb.med.umn.edu/). The large deletion in the *TYR *gene was first reported by Schnur et al. in 1996 in an OCA1B patient who had compound heterozygous mutations of a *TYR *gene [18, 19]. Further, Sun et al. reported 12 *TYR *gene mutations in a total of 10 patients with OCA1. However, none of these studies have reported co-inheritance of OCA 1B and ID. In our study, we have identified a rare homozygous deletion of exon 3 in the *TYR *gene. To the best of our knowledge, this is the first case of large deletion of exon 3 in the *TYR *gene from the Indian population. This mutation has not been reported in any human mutation databases, including the albinism database so far. However, exon 3 deletion was first described by Mauri et al. in a single Turkish family affected with albinism [20]. The *TYR *gene exon 3 region encodes a copper B binding region bound by three conserved histidine residues, stimulating the tyrosinase enzyme activity. Tyrosinase protein encoded by the *TYR* gene is a copper monooxygenase that catalyzes the hydroxylation of tyrosine to dopaquinone in the melanin biosynthetic pathway and oxidation of L-DOPA [10]. In our case, reduced melanin synthesis is due to homozygous deletion of exon 3 of the *TYR* gene leading to inactivation of the tyrosinase enzyme. Blaszczyk et al. reported a case with exon 4 deletion in a non-human mammal, albino ferrets (Mustela Putorius Furo), which was similarly affected with hypo-pigmentation [21]. However, multiple exonic deletions have been reported in OCA1B subjects (http://albinismdb.med.umn.edu/). Our extensive literature search revealed that the different mutations within the tyrosinase-coding region causes OCA1 disorders of varying severity [22]. In the present study, whole exon 3 of the *TYR* gene is deleted in siblings inherited through autosomal recessive inheritance. Hence homozygous recessive genes transmitted through parental consanguinity have a high risk of developing genetic diseases.

The Retinitis Pigmentosa (RP) is a genetic disorder of the eye that leads to decreased peripheral vision where night blindness is caused by mutation in 50 genes including RP1 gene [23, 24]. In our study, a heterozygous RP1 gene variation (p.Gln1546Ter) was identified in all three children, mother and maternal grandfather. The *RP1* gene variants identified in the family may not be associated with the phenotype due to the haploinsufficiency of the NDK isoenzyme [25]. However, follow-up of these patients is important to assess the future development of the RP.

The genetic basis of ID, multiple congenital anomalies (MCA), and an autism spectrum disorder (ASD) in children is not completely understood. However, CNVs identified by array-CGH have been reported in children affected with ID [26]. The co-occurrence of ID and OCA is a rare clinical presentation and diagnosis is challenging through clinical evaluation. We have studied CNVs in the affected siblings and their parents to understand the molecular basis of ID. The 10q23.2-> 10q23.31 deletion identified in our study is inherited by the 1^st^ child from his father. Chromosome 10q23 contains more than five OMIM genes (*PTEN, KLLN, ACTA2, FAS, LIPA, and SLC16A12*). This chromosomal region is associated with several conditions, including ID (Additional file 1: Table S2). In the present study, the father and the child had a history of delayed development and poor social behavior, which may be associated with the deletion of chromosomal 10q23.2-> 10q23.31 region. According to the literature, the PTEN gene resides on 10q23.2-> 10q23.31 chromosomal region and is reported to be associated with neurological functions [27, 28]. The subjects with *PTEN *gene mutations have a high risk of autism spectrum disorder, macrocephaly, and cognitive deficits [29, 30]. The additional clinical presentation (ID) in the first child may be due to the loss of multiple genes, including the *PTEN* gene in the 10q23.2-> 10q23.31 region. The SLITRK1 and SLITRK6 genes located on 13q31.1-> 13q31.3 region are known to be associated with ID, ADHD, and facial dysmorphism [31]. Hence a denovo duplication (7.6 MB) of chromosome 13q31.1-> 13q31.3 region identified in the third child may be associated with ID. The duplication of 13q has also been reported in cases with autistic behavior, developmental delay, dysmorphism, including broad thumbs, strabismus, trigonocephaly (Additional file 1: Table S3).

The *SLITRK1* gene is located in the 13q31.1 region and is associated with Gilles de la Tourette's syndrome (OMIM-137580). The individuals affected with these CNVs generally present with hyperactivity, impulsivity, repetitive movements [32]. However, more critical investigation of the genes involved in the duplication can provide precise genotype–phenotype correlation. Though paternally inherited deletion of 15q11.1-> 11.2(Chr coordinates: 20,575,646–22,784,582) present in all three siblings, the female sibling has no presentation of ID. Hence it indicates that 15q11.1-> 11.2 deletion may not be associated with ID. Genotype–phenotype correlation revealed that the exon3 deletion of the *TYR* gene is associated with the phenotype of OCA1B, and additional clinical presentation (ID) may be due to co-segregation of pathogenic CNVs in the family. Therefore, molecular evaluation is important for appropriate genetic counseling and management of the disease.

## Conclusion

The OCA1B phenotype in children is due to the autosomal recessive inheritance of a rare exon 3 deletion of the *TYR* gene, which was identified through systemic molecular strategy. The additional clinical presentation (ID) may be due to the CNVs identified by array CGH. Therefore, a combination of genetic approaches is essential in cases with a complex phenotypic presentation for early diagnosis and better disease management.

## Supplementary Information

**Additional file 1. Table S1:** List of Genes associated with OCA and *RP1* tested through NGS. **Table S2:** Clinical features of patients associated with deletion of chromosome 10q region. **Table S3:** Clinical features of patients associated with duplication of chromosome 13q region..

## Data Availability

The human reference genome (GRCh37/hg19) (http://www.ensembl.org/) was used for the interpretation of genomic data. The project data is deposited under BioProject database with accession ID: PRJNA776081 (https://www.ncbi.nlm.nih.gov/bioproject/776081). Biosample IDs for all the participants are SAMN22713530, SAMN22713529, SAMN22713528, SAMN22713527, SAMN22713526, SAMN22705527, SAMN22696442 linked to BioProject PRJNA776081 (https://www.ncbi.nlm.nih.gov/bioproject/776081). The variants identified in TYR and RP1 gene are submitted to ClinVar under accession IDs SCV001448215 cited as “National Center for Biotechnology Information. ClinVar; [VCV000992661.1], https://www.ncbi.nlm.nih.gov/clinvar/variation/VCV000992661.1 (accessed Dec. 2, 2021)” and SCV002003955 cited as “National Center for Biotechnology Information. ClinVar; [VCV001315572.1], https://www.ncbi.nlm.nih.gov/clinvar/variation/VCV001315572.1 (accessed Dec. 2, 2021) respectively”. The details of NGS data is submitted to NCBI Sequence Read Archive under accession ID SRX13085209 (https://www.ncbi.nlm.nih.gov/sra?linkname=bioproject_sra_all&from_uid=776081) / SRP345279 (https://trace.ncbi.nlm.nih.gov/Traces/sra/?study=SRP345279) / SRR16892488 (https://trace.ncbi.nlm.nih.gov/Traces/sra/?run=SRR16892488). The RP1 gene sequences are submitted to GeneBank through BankIt under submission ID 2,515,543, the accession numbers are OL351360—https://www.ncbi.nlm.nih.gov/nuccore/OL351360. OL351361—https://www.ncbi.nlm.nih.gov/nuccore/OL351361. OL351362—https://www.ncbi.nlm.nih.gov/nuccore/OL351362. OL351363—https://www.ncbi.nlm.nih.gov/nuccore/OL351363. OL351364—https://www.ncbi.nlm.nih.gov/nuccore/OL351364. OL351365—https://www.ncbi.nlm.nih.gov/nuccore/OL351365. The array CGH data is submitted to GEO data base under project ID PRJNA785601 (https://www.ncbi.nlm.nih.gov/bioproject/PRJNA785601); with accession # GEO: GSE190026 (https://www.ncbi.nlm.nih.gov/geo/query/acc.cgi?acc=GSE190026).

## Abbreviations

*TYR*: Tyrosinase
*RP1*: Retinitis pigmentosa 1
OCA 1: Occulocuteneous albinism 1
ID: Intellectual disability
MLPA: Multiplex ligation-dependent probe amplification
NGS: Next-generation sequencing
CNVs: Copy number variations
CGH: Comparative genomic hybridization
HPS: Hermansky–Pudlak syndrome
CHS: Chediak–Higashi syndrome
GS: Griscelli syndrome
CMBS: Cross-McKusick-Breen syndrome
ADHD: Attention deficit-hyperactivity disorder
BERA: Brainstem evoked response audiometry
TES: Targeted exome sequencing
SS: Sanger sequencing
OMIM: Online mendelian inheritance in man
PCR: Polymerase chain reaction
ACMG: American College of Medical Genetics
HGMD: Human gene mutation database
MCA: Multiple congenital anomalies
ASD: Autism spectrum disorder
