# Correction to: Novel deletion of exon 3 in *TYR* gene causing Oculocutaneous albinism 1B in an Indian family along with intellectual disability associated with chromosomal copy number variations

**DOI:** 10.1186/s12920-022-01159-2

**Published:** 2022-01-18

**Authors:** Somprakash Dhangar, Purvi Panchal, Jagdeeshwar Ghatanatti, Jitendra Suralkar, Anjali Shah, Babu Rao Vundinti

**Affiliations:** Department of Cytogenetics, National Institute of Immunohaematology (ICMR), 13th Floor, New Multistoried Building, K.E.M Hospital Campus, Parel, Mumbai, 400012 India

## Correction to: BMC Medical Genomics (2022) 15:2 10.1186/s12920-021-01152-1

Following publication of the original article [1], the authors identified an error in Fig. 1b. In the figure, the label of the Carrier parents is typed as “TYR Exon 3 homozygous deletion” which is incorrect and should be “TYR Exon 3 heterozygous deletion”. The corrected Fig. 1b is supplied in this correction article.

**Fig. 1:**
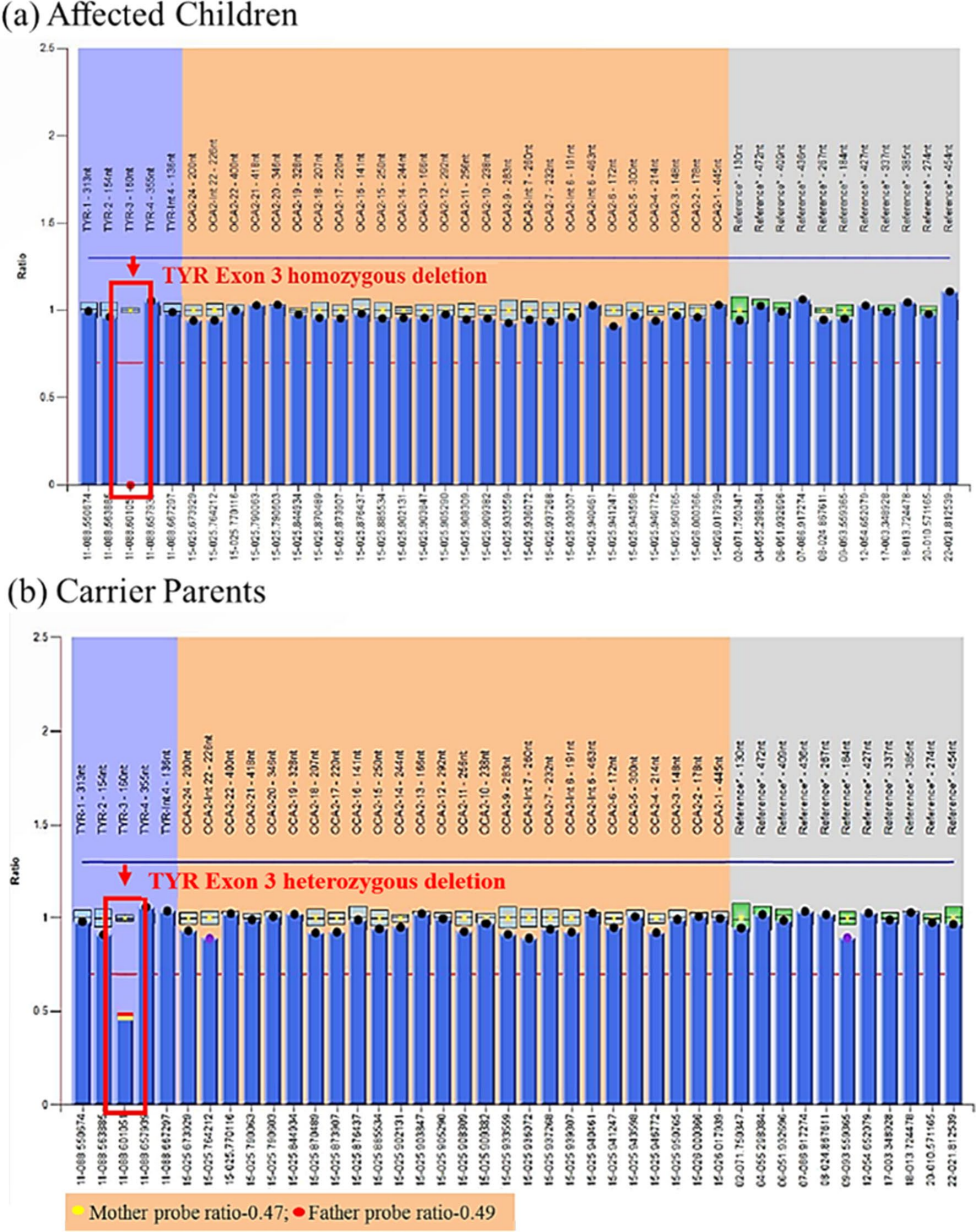
MLPA results showing TYR exon 3 deletion: (**a**) All 3 children of fourth-generation with probe ratio 0.00 (**b**) Carrier parents (Mother with probe ratio 0.47 and father with probe ratio 0.49)

Further to this, the authors identified words in the text which are merged. The authors apologize for the inconvenience. The original article [1] has been corrected.
