# Supplementary material for: Novel deletion of exon 3 in TYR gene causing Oculocutaneous albinism 1B in an Indian family along with intellectual disability associated with chromosomal copy number variations
Source: BMC Med Genomics. 2022 Jan 3;15:2. doi: 10.1186/s12920-021-01152-1 (PMC8722050; doi:10.1186/s12920-021-01152-1)
Supplement: Supplementary file 1 — Additional file 1. Table S1: List of Genes associated with OCA and RP1 tested through NGS. Table S2: Clinical features of patients associated with deletion of chromosome 10q region. Table S3: Clinical features of patients associated with duplication of chromosome 13q region.. [file 12920_2021_1152_MOESM1_ESM.docx]

**Table S1.** List of Genes associated with OCA and RP1 tested through NGS

| S. no. | Gene | Percentage of coding region covered |
| --- | --- | --- |
| 1 | *AP3B1* | 100.00 |
| 2 | *BLOC1S6* | 100.00 |
| 3 | *EPG5* | 100.00 |
| 4 | *HPS3* | 100.00 |
| 5 | *HPS6* | 100.00 |
| 6 | *MLPH* | 100.00 |
| 7 | *RAB27A* | 100.00 |
| 8 | *SLC45A2* | 100.00 |
| 9 | *AP3D1* | 100.00 |
| 10 | *C10ORF11* | 96.72 |
| 11 | *GPR143* | 100.00 |
| 12 | *HPS4* | 100.00 |
| 13 | *LYST* | 100.00 |
| 14 | *MYO5A* | 100.00 |
| 15 | *RP1* | 100.00 |
| 16 | *TYR* | 90.69 |
| 17 | *BLOC1S3* | 100.00 |
| 18 | *DTNBP1* | 100.00 |
| 19 | *HPS1* | 100.00 |
| 20 | *HPS5* | 100.00 |
| 21 | *MC1R* | 100.00 |
| 22 | *OCA2* | 100.00 |
| 23 | *SLC24A5* | 100.00 |
| 24 | *TYRP1* | 100.00 |

**Table S2.** Clinical features of patients associated with deletion of chromosome 10q region

| Chr. No. | Cytoband | Deletion/ Duplication | Size  Kb/Mb | Features | Reference |
| --- | --- | --- | --- | --- | --- |
| Chr10 | 10q22.3q23.31 | Deletion | ∼7.2 Mb | **Behavioral** and neurodevelopment abnormalities, including **cognitive impairment**, **autism**, **hyperactivity**, and possibly **psychiatric** disease | [1] |
| Chr10 | 10q22.3- q23.3 | Deletion | N.A. | **Speech** and **language delay**, **mild facial dysmorphism**, cerebellar anomalies, cardiac defects and congenital breast aplasia | [2] |
| Chr10 | 10q23.2q23.33 | Deletion | N.A. | **ID**, **dysmorphic features** and juvenile polyposis coli | [3] |
| Chr10 | 10q23.2– q23.31 | Deletion | 3.05 MB | Macrocephaly, muscle weakness, pigmented macules  of the glans penis, **developmental delay** and chronic diarrhoea, severe form of **BRRS**, including juvenile polyposis of infancy symptoms such as the extent of the polyps along the gastrointestinal tract, early presentation age, protein‐losing enteropathy and failure to thrive. | [4] |
| Chr 10 | 10q23.2 - q23.31 | Deletion | 2.6 MB | Developmental delay, mild facial dysmorphism, ID | Present study |

N.A. Not available, Bolding represents features in common with our patients (1^st^ child and father), Partial anomalies similar to BRRS present in our case (first male child of 4^th^ generation)

1. Balciuniene J, Feng N, Iyadurai K, Hirsch B, Charnas L, Bill BR, Easterday MC, Staaf J, Oseth L, Czapansky-Beilman D, Avramopoulos D. Recurrent 10q22-q23 deletions: a genomic disorder on 10q associated with cognitive and behavioral abnormalities. The American Journal of Human Genetics. 2007 May 1;80(5):938-47..

2. Van Bon BW, Balciuniene J, Fruhman G, Nagamani SC, Broome DL, Cameron E, Martinet D, Roulet E, Jacquemont S, Beckmann JS, Irons M. The phenotype of recurrent 10q22q23 deletions and duplications. European journal of human genetics. 2011 Apr;19(4):400-8..

3. Tsuchiya KD, Wiesner G, Cassidy SB, Limwongse C, Boyle JT, Schwartz S. Deletion 10q23. 2‐q23. 33 in a patient with gastrointestinal juvenile polyposis and other features of a Cowden‐like syndrome. Genes, Chromosomes and Cancer. 1998 Feb;21(2):113-8.

4. Waisbourd‐Zinman O, Mamula P, Piccoli DA. Chromosome 10q23 Deletion Syndrome: An Overlap of Bannayan–Riley–Ruvalcaba Syndrome and Juvenile Polyposis Syndrome. Journal of paediatrics and child health. 2016 Aug;52(8):852-.-.

**Table S3.** Clinical features of patients associated with duplication of chromosome 13q region

| Chr.  No. | Cytoband | Duplication / deletion | Size  Kb/Mb | Features | Reference |
| --- | --- | --- | --- | --- | --- |
| Chr 13 | 13q31.3q32.3 | Micro duplication | 8.2 Mb | **Facial dysmorphism, learning difficulties and autistic spectrum disorder** | [1] |
| Chr 13 | 13q21.33-q34 | Duplication | 46.344  Mb | Hypoxic ischemic encephalopathy, episodes of apnea, **dysmorphic features** and mild **psychomotor retardation**. | [2] |
| Chr 13 | 13q21.31-  13q31.1 | Duplication | 21 Mb | **ID**, **behavioral problems,** seizures, hearing loss, strabismus, dental anomalies, hypermobility, juvenile hallux valgus, and **mild dysmorphic features** | [3] |
| Chr 13 | 13q31.1q31.3 | Duplication | 7.6 Mb | Developmental delay, mild facial dysmorphism,  ID, ADHD | Present study |

Bolding represents features in common with our patient [3^rd^ child (male)]

1. Atack E, Fairtlough H, Smith K, Balasubramanian M. A novel (paternally inherited) duplication 13q31. 3q32. 3 in a 12-year-old patient with facial dysmorphism and developmental delay. Molecular syndromology. 2014;5(5):245-50.
2. Wei Y, Gao X, Yan L, Xu F, Li P, Zhao Y. Prenatal diagnosis and postnatal followup of partial trisomy 13q and partial monosomy 10p: A case report and review of the literature. Case reports in genetics. 2012 Oct 23;2012.
3. Mathijssen IB, Hoovers JM, Mul AN, Man HY, Ket JL, Hennekam RC. Array comparative genomic hybridization analysis of a familial duplication of chromosome 13q: a recognizable syndrome. American Journal of Medical Genetics Part A. 2005 Jul 1;136(1):76-80.
